# Familienrekonstitution und Online-Genealogien zur Analyse der geschlechtsspezifischen differenziellen Sterblichkeit im historischen Kontext

**DOI:** 10.1007/s00103-024-03865-x

**Published:** 2024-04-15

**Authors:** Robert Stelter

**Affiliations:** 1 Wirtschaftswissenschaftliche Fakultät, Universität Basel, Peter Merian-Weg 6, 4002 Basel, Schweiz; 2 Max-Planck-Institut für Demografische Forschung, Konrad-Zuse-Str. 1, 18057 Rostock, Deutschland

**Keywords:** Lebenserwartung, Online-Genealogien, Familienrekonstitution, Differenzielle Mortalität, Verzerrungen, Life expectancy, Online genealogies, Family reconstitution, Differential mortality, Biases

## Abstract

**Hintergrund:**

Familienrekonstitutionen und Familienstammbäume genealogischer Online-Plattformen sind 2 mögliche Datenquellen für die Untersuchung der Sterblichkeit in einer Zeit, als noch keine amtlichen Sterbetafeln verfügbar waren. Der vorliegende Artikel diskutiert anhand zweier Beispiele, der Familienrekonstitution aus Imhof und dem auf *geni.com* beruhenden Datensatz *FamiLinx*, die geschätzten Verläufe der Lebenserwartung im Deutschen Reich mit einem Fokus auf die geschlechtsspezifische differenzielle Mortalität.

**Methoden:**

Mithilfe der Individualdaten aus der Familienrekonstitution und aus den Online-Genealogien werden die geschlechtsspezifischen Sterbetafeln geschätzt. Aus ihnen wird die bedingte Lebenserwartung ermittelt und die entsprechende geschlechtsspezifische differenzielle Mortalität abgeleitet und mit den amtlichen Sterbetafeln für die Jahre 1871–1910 abgeglichen. Der Beitrag der einzelnen Altersklassen zur differenziellen Sterblichkeit wird mit dem *Stepwise Replacement Algorithm* bestimmt.

**Ergebnisse:**

Die Ergebnisse der Familienrekonstitution überschätzen die Lebenserwartung nach 1871 weniger stark als die FamiLinx-Schätzungen. Die geringere Sterblichkeit der Frauen in der amtlichen Statistik wird von beiden Quellen nicht abgebildet. Im Gegensatz zur amtlichen Statistik ist die geschätzte Lebenserwartung der Männer höher als die der Frauen. Diese verzerrte geschlechtsspezifische Abbildung der Mortalitätsraten geht insbesondere auf die Altersklassen von 15 bis 45 Jahren zurück.

**Diskussion:**

Der *Notability Bias*, der patriarchische Ansatz in der Erstellung von Familienstammbäumen und die Müttersterblichkeit sind mögliche Ursachen für diese Beobachtungen in FamiLinx. In der Familienrekonstitution ist die mit der Mobilität einhergehende Zensierung ein Erklärungsansatz.

## Abstract (en)

**Background:**

Family reconstitution and data from online genealogies, such as *FamiLinx*, are two potential sources for investigating mortality dynamics for the period before official lifetables became available. In this paper, we use two of them, the family reconstitution of Imhof and the *FamiLinx* dataset based on *geni.com*, to estimate dynamics in life expectancy and discuss the sex-specific differential mortality in the German Empire.

**Method:**

Sex-specific lifetables are estimated for the territory of the German Empire from the individual data of the family reconstitution and the online genealogies. On the basis of these lifetables, we estimate the conditional life expectancy and derive the corresponding sex-specific differential mortality. Findings are compared with the official lifetable of the German Empire in 1871–1910. The contribution of each age group to the differential mortality is determined using the stepwise-replacement algorithm.

**Results:**

The family reconstitution overestimates conditional life expectancy less than FamiLinx after 1871, when official lifetables are available in the German Empire. However, both sources fail to capture the sex-specific mortality differentials of the official lifetables at the end of the nineteenth century and show a higher life expectancy for males instead of females. The bias in sex-specific mortality rates is particularly pronounced in the age groups 15 to 45.

**Discussion:**

Finally, we discuss possible explanations for the biased findings. Notability bias, the patriarchal approach to family trees, and maternal mortality are important mechanisms in the FamiLinx dataset. Censoring due to mobility serves as a potential reason for the bias in the family reconstitution.

## Einleitung

Seit der industriellen Revolution reduziert sich die Sterblichkeit der Menschen in einem beträchtlichen Ausmaß. Um die langfristige Sterblichkeitsentwicklung zu verstehen, rücken Datenquellen fernab der amtlichen Sterbetafeln ins Blickfeld. Standardwerke wie Wrigley et al. [1], Henry und Fleury [2] oder Imhof [3] bedienen sich der Methode der Familienrekonstitution (Family Reconstitution), bei der die Daten aus Kirchenbüchern und anderen historischen Quellen familienweise zusammengestellt werden. Sie erlauben die Untersuchung des Einsetzens der systematischen Verbesserungen in der Sterblichkeit und die Herausarbeitung der differenziellen Mortalität entlang unterschiedlicher Dimensionen wie Geschlecht, sozialem Status oder Urbanisierung für die Zeit vor den ersten amtlichen Sterbetafeln. Im Falle des Deutschen Reiches wurden diese vom statistischen Reichsamt erstmals für den Zeitraum 1871–1880 publiziert.

Kaplanis et al. [4] bringen mit Online-Ahnensterbetafeln („crowdsourced online genealogies“) eine innovative Alternative zur Familienrekonstitution in die historische Demografie ein und ebnen damit gleichzeitig der Big-Data-Revolution in der historischen Demografie den Weg. In ihrem *FamiLinx*-Datensatz sind mehr als 86 Mio. öffentliche Profile von geni.com, einer der größten kooperativ aufgebauten genealogischen Plattformen, für die wissenschaftliche Auswertung aufbereitet. Diese neuen Individualdaten bieten der Wissenschaft eine Alternative oder Ergänzung zur Familienrekonstitution und erlauben neue Perspektiven (siehe beispielsweise Black et al. [5] zur Korrelation in der Sterblichkeit zwischen den Generationen oder Clark [6] zur Vererbung des sozialen Status). Gleichzeitig beinhalten sie aber auch neue Herausforderungen – insbesondere im Hinblick auf die Gefahr, verzerrte Ergebnisse zu liefern.

Im vorliegenden Artikel werden mögliche Verzerrungen in der Sterblichkeitsanalyse basierend auf Daten, die aus der Ahnenforschung gewonnen wurden, diskutiert. Im Fokus steht dabei die geschlechtsspezifische differenzielle Mortalität im Gebiet des Deutschen Reiches. Dafür werden Mortalitätsschätzungen aus Online-Familienstammbäumen (FamiLinx) der Familienrekonstitution aus Imhof [3] gegenübergestellt und mit den amtlichen Statistiken – sobald verfügbar – abgeglichen. Damit trägt der Artikel zu 2 Literatursträngen bei. Aus methodischer Perspektive wird ein Beitrag zur aktuellen kontroversen Diskussion über die Sterblichkeitsbetrachtungen mithilfe von FamiLinx (oder kooperativ aufgebauten Online-Genealogien im Allgemeinen) geleistet. Kaplanis et al. [4] und Blanc [7] ziehen im Hinblick auf die Repräsentativität des FamiLinx-Datensatzes nach einem Abgleich mit Sterbetafeln der *Human Mortality Database* ein positives Fazit. Letzterer betont gar die Vorteile gegenüber den Familienrekonstitutionen à la Wrigley et al. [1]. Andere Autoren, wie Stelter und Alburez-Gutierrez [8] oder Chong et al. [9], sind weniger optimistisch und weisen auf die potenziellen Verzerrungen in der Mortalität hin [8] oder schlagen Korrekturmethoden für Mortalitätsverzerrungen vor [9].

Gleichzeitig reiht sich der Artikel in eine Reihe von Arbeiten zur langfristigen Sterblichkeitsentwicklung ein. Neben den bereits genannten auf der Familienrekonstitution basierenden Werken sind dies insbesondere Publikationen, die spezifische Teilbevölkerungen untersuchen. Mit einem Fokus auf Kardinäle [10], Wissenschaftler [11, 12] oder Mediziner [13] ist eine Untersuchung der geschlechtsspezifischen differenziellen Mortalität dabei häufig aufgrund der geringen Fallzahlen bei Frauen nicht möglich. In ihren Untersuchungen zu berühmten Persönlichkeiten können de la Croix und Licandro die Geschlechterdimension zwar berücksichtigen, müssen das Geschlecht aber über Vornamen approximieren. Zudem ist der Frauenanteil auch hier noch sehr gering, sodass die differenzielle Mortalität der Geschlechter nicht im Kern der Betrachtung steht [14]. Mit einem Fokus auf ausgewählte Berufsgruppen wird eine Analyse der geschlechtsspezifischen Mortalität für bildende Künstler beispielsweise ab 1750 möglich [15]. Bei der Betrachtung von Adeligen hingegen bildet sie ein wichtiges Merkmal [16]. All diese Arbeiten nutzen die Vorteile der guten Datenverfügbarkeit für die entsprechenden Bevölkerungsgruppen. Ein Rückschluss auf die Allgemeinbevölkerung und ihre differenzielle Mortalität wird damit aber nicht angestrebt. Der vorliegende Artikel untersucht daher explizit die geschlechtsspezifische differenzielle Sterblichkeit in der historischen Allgemeinbevölkerung. Neben der Darlegung ihrer Ausgestaltung wird kritisch hinterfragt, ob die beiden untersuchten Individualdatensätze die Mortalitätsunterschiede tatsachengetreu abbilden können. Dafür werden Schätzungen auf Grundlage von Online-Genealogien und Familienrekonstitution mit den ersten amtlichen Statistiken abgeglichen.

## Methoden

### Daten

Für die Analyse werden 3 verschiedene Datenquellen herangezogen: Als Referenzwerte dienen die 4 abgekürzten Sterbetafeln des Deutschen Reiches 1871–1880, 1881–1890, 1891–1900 und 1901–1910 [17]. Diese liegen geschlechtsspezifisch in 5er-Altersgruppen vor und wurden in digitaler Form der Human *Life-Table Database* (www.lifetable.de) entnommen.

**Figure 1:**
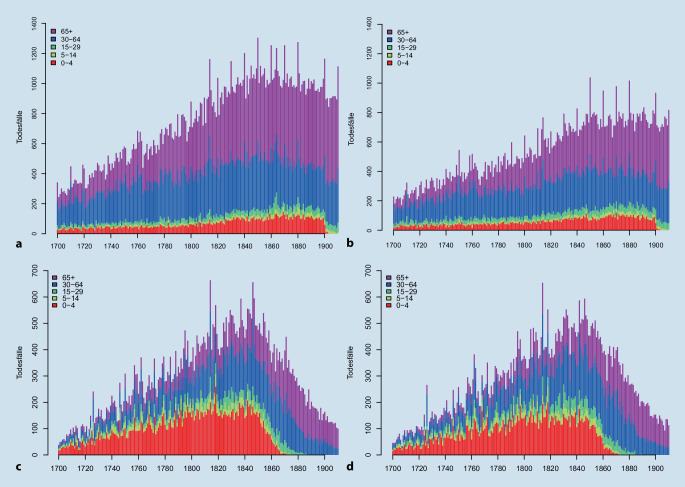

Die Daten der kooperativ aufgebauten Familienstammbäume entstammen den ca. 86 Mio. öffentlichen Profilen der Online-Plattform *geni.com*, die Kaplanis et al. im FamiLinx-Datensatz aufbereitet haben [4]. Diese werden auf die Profile beschränkt, die sich entsprechend der demografischen Ereignisse Geburt und/oder Tod dem Territorium des Deutschen Reiches zuschreiben lassen (siehe Stelter und Alburez-Guiterrez [8]). So verbleibt ein Sample von 273.945 Sterbefällen zwischen 1700 und 1910, für die das Sterbealter berechnet werden kann. Von diesen entfallen ca. 115.000 Profile auf Frauen und ca. 159.000 auf Männer. Die geschlechts- und altersspezifische zeitliche Verteilung der Sterbefälle kann Abb. 1 entnommen werden. Neben der bekannten Problematik einer Untererfassung von Sterbefällen in der Altersklasse 0 bis unter 5 Jahre fällt hier ein Bruch in dieser Altersklasse Anfang des 20. Jahrhunderts auf. Ab 1904 sind bei beiden Geschlechtern keine Sterbefälle in der Altersklasse 0 bis unter 5 Jahre mehr erfasst.

Die Vergleichsdaten der Familienrekonstitution entstammen dem Werk von Imhof [3]. Sie umfassen insgesamt 51 Orte für Westdeutschland, die aus Ortsfamilienbüchern in den ländlichen Regionen Ostfriesland, Hartum, Schwalm, Saarland, Herrenberg und der Ortenau aufbereitet wurden. Zusätzlich werden Daten für Hamburg aus Geschlechterbüchern erfasst. Damit beschränken sich die Daten auf Orte aus dem Gebiet der Bundesrepublik Deutschland vor der Wiedervereinigung mit insgesamt 60.000 weiblichen und 62.000 männlichen Sterbefällen zwischen 1700 und 1910, für die das Sterbealter berechnet werden kann. Bei der Verteilung der Sterbefälle über das Alter, das Geschlecht und die Zeit in Abb. 1 wird der deutlich größere Anteil der Sterbefälle im Säuglings- und Kleinkindalter sichtbar. Auch bei den Kindern und jungen Erwachsenen ist der Anteil höher. Gleichzeitig illustriert Abb. 1 das Auslaufen der Erfassung von Personen jüngerer Geburtskohorten in der zweiten Hälfte des 19. Jahrhunderts in den Ortsfamilienbüchern. Ein Vergleich zu den Sterbetafeln des Deutschen Reiches wird damit zusätzlich erschwert.

### Datenanalyse

Für die Analyse der Sterblichkeit wird der *direkten* Methode gefolgt. Auf eine Verwendung von Modellsterbetafeln, wie sie im Zuge der *indirekten* Methodik in der historischen Demografie oft Anwendung fand, wird damit verzichtet [18]. Vielmehr wird sich auf die Individuen beschränkt, für die Geburts- und Sterbejahr in den jeweiligen Daten vorliegen. Eine Korrektur der Risikobevölkerung für rechtszensierte Fälle, zum Beispiel durch Abwanderung, erfolgt nicht.^1^ Ausgehend von der so ermittelten Risikobevölkerung und den Sterbefällen beruht die Analyse auf demografischen Standardtools (siehe auch Stelter und Alburez-Guiterrez [8]). Sterbeereignisse und durchlebte Personenjahre werden zunächst in rollenden 10-Jahres-Intervallen aggregiert. Der Verweis auf ein spezifisches Jahr markiert nachstehend immer den Beginn des 10-Jahres-Intervalls.^2^ Anschließend werden mithilfe der aggregierten Sterbefälle und der Risikobevölkerungen die Sterberaten ermittelt und mithilfe von *P‑Splines* entlang der Dimensionen Alter und Zeit geglättet [19]. Basierend auf den geglätteten Sterberaten werden dann Sterbetafeln berechnet. Um den Einfluss der auslaufenden Daten in der Familienrekonstitution zum Ende des 19. Jahrhunderts, wenn die Referenzwerte aus der amtlichen Statistik vorliegen, zu begrenzen, wird der Fokus auf die adulte Lebenserwartung im Alter von 30 Jahren gelegt. Die Schätzung der Konfidenzintervalle wird durch Monte-Carlo-Simulationen vorgenommen [20]. Zusätzlich erfolgt eine Dekomposition der Beiträge einzelner Altersklassen zur differenziellen Mortalität zwischen den Geschlechtern mithilfe des *Stepwise Replacement Algorithm* [21, 22].

## Ergebnisse

### Bedingte Lebenserwartung im Alter von 30 Jahren

**Figure 2:**
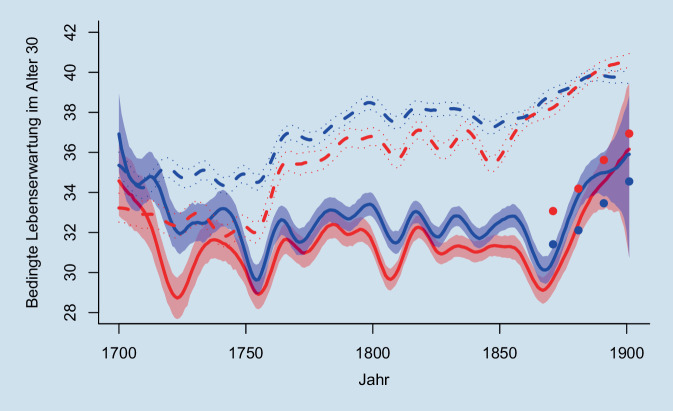

Abb. 2 zeigt die bedingte geschlechtsspezifische Lebenserwartung im Alter von 30 Jahren für die Referenzwerte aus den amtlichen Sterbetafeln sowie die Schätzungen entsprechend den Familienstammbäumen von geni.com (FamiLinx) und der Familienrekonstitution (Imhof). Die Unterschiede zu Beginn des 18. Jahrhunderts für die beiden verfügbaren Datenquellen fallen relativ gering aus bzw. lassen sich aufgrund der fallzahlbedingten statistischen Unsicherheiten nur bedingt interpretieren. Während sich bis ca. 1850 keine systematische Verbesserung in der aus der Familienrekonstitution geschätzten Lebenserwartung ergibt, kommt es nach 1750 zu einem Anstieg der adulten Lebenserwartung in der Schätzung mithilfe von FamiLinx. Dadurch ergibt sich für die nächsten 100 Jahre ein Unterschied von ca. 4,9 Jahren in der Lebenserwartung der Frauen und 5,2 Jahren in jener der Männer. Ab ca. 1850 setzt dann die systematische, anhaltende Sterblichkeitsverbesserung in FamiLinx ein. Um ca. 2 Jahrzehnte verzögert folgt die kontinuierliche Reduktion der Sterblichkeit in der auf der Familienrekonstitution basierenden Schätzung.

Im Jahr 1871, in dem der Abgleich mit den amtlichen Sterbetafeln des statistischen Reichsamtes erstmals möglich ist, weicht die bedingte männliche Lebenserwartung im Alter von 30 Jahren (für Westdeutschland) in der Familienrekonstitution lediglich um 0,85 Jahre von den amtlichen Werten im gesamten Deutschen Reich ab. Während die Lebenserwartung in der amtlichen Statistik zu diesem Zeitpunkt noch niedriger ist, ist sie in den folgenden Jahren bis zu 1,7 Jahre höher. Für Frauen beträgt diese Differenz immerhin schon ca. 3,4 Jahre im Jahr 1871, bleibt aber bis Anfang des 20. Jahrhunderts positiv zugunsten der amtlichen Statistik. Wie bereits in Stelter und Alburez-Gutierrez [8] gezeigt, ist diese Differenz für Schätzungen mithilfe der aus den FamiLinx-Daten ermittelten Sterbetafel positiv zugunsten von FamiLinx und deutlich höher. Sie beträgt 5,2 Jahre für die bedingte Lebenserwartung der Frauen und 7,5 Jahre für jene der Männer.

Neben den unterschiedlichen Ergebnissen im Hinblick auf das Niveau und die Dynamik in der Entwicklung der Sterblichkeit beleuchtet Abb. 2 auch die geschlechtsspezifische differenzielle Mortalität. Die Lebenserwartung entsprechend den Sterbetafeln des statistischen Reichsamtes dokumentiert die bekannten Sterblichkeitsvorteile von Frauen, wie sie auch heute noch in der Bundesrepublik Deutschland vorliegen [23]. 30-jährige Frauen leben im Durchschnitt zwischen 1,7 Jahren im Jahr 1871 und 2,4 Jahren im Jahr 1901 länger. Diesen Sterblichkeitsvorteil finden wir (signifikant) nur ganz zum Ende des Beobachtungsfensters für die aus FamiLinx geschätzte Lebenserwartung im Alter 30 in Abb. 2 und dies auch nur in sehr viel geringerem Umfang. Vor der Mitte der 1880er-Jahre haben Männer tendenziell eine höhere Lebenserwartung und vor 1870 ist dieser Unterschied durchgehend signifikant. Ein ähnliches Bild zeichnet sich für die Familienrekonstitution. Zum Ende des 19. Jahrhunderts liegen beide Geschlechter – bei erheblicher Unsicherheit durch die Datenbeschaffenheit – gleich auf, während Männer in den Jahren vorab eine höhere Lebenserwartung genießen.

### Differenzielle Sterblichkeit von Frauen und Männern

**Figure 3:**
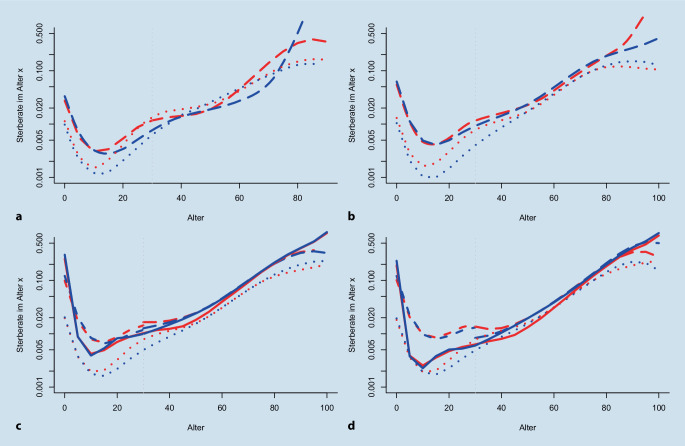

Die geschätzten adulten Lebenserwartungen, sowohl aus FamiLinx als auch mithilfe der Familienrekonstitution aus Imhof, bilden die geschlechtsspezifische differenzielle Mortalität der amtlichen Sterbetafeln nicht ab. Um die Verzerrung genauer zu beleuchten, zeigt Abb. 3 die altersspezifischen Sterberaten zu 4 Zeitpunkten 1700–1709, 1800–1809, 1871–1880 und 1881–1890. Die amtlichen Sterbetafeln in den Jahren 1871–1880 und 1881–1890 weisen relativ kleine Unterschiede in der geschlechtsspezifischen Mortalität bis zum Alter von 35 Jahren auf. Im Anschluss ist die männliche Sterblichkeit höher und begründet die dokumentierte differenzielle Sterblichkeit mit einer höheren Lebenserwartung der Frauen in Abb. 2. Zum Vergleich: In der aktuellen Sterbetafel 2018–20 ergibt sich die Differenz in der altersspezifischen Sterblichkeit bereits nach einem Alter von 15 Jahren [23].

Diese klare Differenz in der bedingten Lebenserwartung der amtlichen Statistik des ausgehenden 19. Jahrhunderts für das Deutsche Reich zugunsten der Frauen findet sich weder in FamiLinx noch in der Familienrekonstitution zu beiden Zeitpunkten wieder. Bis zum Alter von 15 Jahren dokumentieren wir ebenfalls keine klaren Unterschiede in der differenziellen Mortalität. Lediglich die bekannte Untererfassung der Sterblichkeit im Säuglings- und Kindesalter wird in beiden Individualdatensätzen sichtbar. In der Familienrekonstitution beschränkt sich dieses Phänomen auf die Kleinkinder – im Anschluss kommt es gar zu einer Überschätzung der Sterberaten. Allerdings muss hier insbesondere die Einschränkung der Daten für junge Altersklassen aufgrund der auslaufenden Datenerfassung berücksichtigt werden (Abb. 1). Daher wurden die Sterberaten ab dem Alter 30 der Glättung mit den Altersklassen 30–100 entnommen und nur die jüngeren Altersklassen einer Glättung vom Alter 0–100. Der geringe Bruch im Alter von 30 Jahren in Abb. 3c zeigt, dass die auslaufenden Ortsfamilienbücher in den Sterberaten 1871–1880 noch relativ geringe Auswirkungen haben. In 1881–1890 sieht dies anders aus; insbesondere für die Männer kommt es zu einem klaren Bruch.

Im Vergleich zur Familienrekonstitution kommt es in FamiLinx zu einer ausgeprägten Unterschätzung der Säuglings- und Kleinkindersterblichkeit, die bis ins Erwachsenenalter anhält, wenn auch in abgeschwächter Form. In den mittleren Altern bzw. dem fertilen Alter der Frau zeigt Abb. 3 hingegen klare Mortalitätsunterschiede. Frauen weisen in den Altersklassen von 15–45 eine deutlich höhere Sterblichkeit in 1871–1880 und 1881–1890 auf als Männer. Dieses Phänomen lässt sich auch in den Sterbetafeln 1700–1709 und 1800–1809 beobachten. Es scheint damit also in den Daten der Familienrekonstitution und in FamiLinx permanent eine differenzielle Sterblichkeit in den mittleren Altersklassen zu geben, die sich in den amtlichen Statistiken so nicht findet.

**Figure 4:**
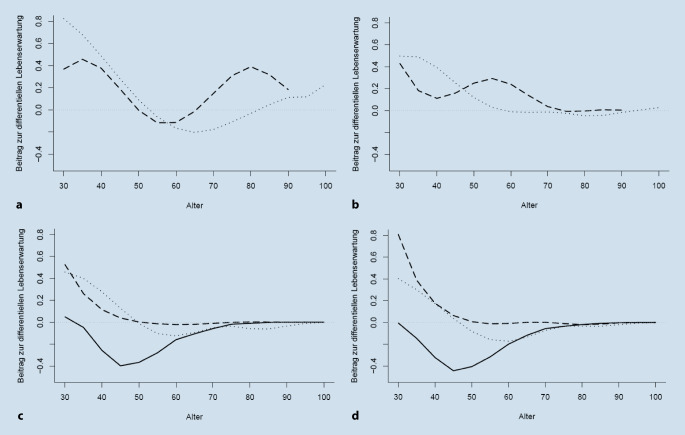

Abb. 4 bildet den Beitrag der einzelnen Altersklassen zum Unterschied zwischen Männern und Frauen in der bedingten Lebenserwartung im Alter 30 ab. Die negativen Werte bis zur Altersklasse 75–79 in der amtlichen Statistik dokumentieren die geringere Sterblichkeit von Frauen in den entsprechenden Altersklassen, wie sie auch der Abb. 3 zu entnehmen ist. Der größte Beitrag zur differenziellen Lebenserwartung lässt sich dabei sowohl in der Sterbetafel 1871–1880 als auch in 1881–1890 der Altersklasse 45–49 mit über 0,4 Jahren zuschreiben. Sowohl in FamiLinx als auch der Familienrekonstitution findet sich ein grundsätzlich anderes Bild. Hier leisten die Altersklassen 30–34 den größten Beitrag – allerdings mit entgegengesetztem, positivem Vorzeichen. In der Familienrekonstitution sind dies rund 0,5 bzw. 0,8 Jahre, bevor der Beitrag mit steigendem Alter gegen 0 strebt. Der positive Beitrag in FamiLinx ist mit jeweils 0,4 Jahren deutlich geringer, sinkt dann ins Negative und konvergiert zur Altersklasse 60–64 zum Verlauf der amtlichen Statistik.

Die altersspezifischen Beiträge zur differenziellen Lebenserwartung in 1700–1709 und 1800–1809 unterscheiden sich deutlich, wie Abb. 4 dokumentiert. Auffällig ist auch hier der große, positive Beitrag der Altersklassen 30–34 und 35–39 in Abb. 4a, b.  In FamiLinx beobachten wir dann einen u‑förmigen Verlauf mit einem Minimum von rund −0,2 Jahren im Alter 65–69 in 1700–1709. In 1800–1809 hingegen laufen die Beiträge zur Altersklasse 55–59 gegen 0 und sind dann vernachlässigbar. Auch in der Familienrekonstitution ergibt sich kein einheitliches Bild. Während der wellenförmige Verlauf in 1700–1709 seine Höhepunkte in den Altersklassen 35–39 und 80–84 hat und sich das Minimum im Alter 55–59 befindet, verschieben sich die lokalen Maxima in die Altersklassen 30–34 und 55–59 in 1800–1809. Das lokale Minimum ist mit der Altersklasse 40–44 deutlich früher und nun positiv.

## Diskussion

Online-Genealogien bieten eine Alternative zur traditionellen Familienrekonstitution für die Schätzung der historischen, geschlechtsspezifischen Sterblichkeit. Die kritische Einordnung der Erkenntnisse aus beiden Alternativen erfordert die Diskussion der externen Validität und der Generalisierbarkeit der Ergebnisse. Dafür bietet sich die Kreuzvalidierung beider Individualdatensätze einerseits sowie der Abgleich mit den amtlichen Statistiken andrerseits an. Abweichende Schätzungen beider Datensätze im Vergleich zu den Werten der amtlichen Sterbetafeln offenbaren systematische Verzerrungen. Diese sind im Wesentlichen auf Selektionsmechanismen in den verfügbaren Daten zurückzuführen. Zusätzlich verursachen Zensierungen Informationsbias.

Die systematischen Verzerrungen können in 4 wesentlichen Erkenntnissen zusammengefasst werden: *(i)* Die geschätzte adulte Lebenserwartung in der Familienrekonstitution wird weniger überschätzt als in FamiLinx, *(ii)* FamiLinx und die Familienrekonstitution bilden die geschlechtsspezifische differenzielle Mortalität nicht tatsachengetreu ab, *(iii)* die verzerrte geschlechtsspezifische Abbildung der Mortalitätsraten Ende des 19. Jahrhunderts fällt insbesondere auf die Altersklassen 15–45 zurück und *(iv)* die höheren Alter werden in FamiLinx weniger verzerrt abgebildet als in der Familienrekonstitution.

Die Ursachen hinter diesen 4 Ergebnissen dürften vielschichtig sein. Zum einen gilt es, die allgemeine Abbildung der Risikobevölkerung und Sterbefälle hinsichtlich der generellen Verzerrungen zu betrachten. Zahlreiche Selektionen bestimmter Bevölkerungsgruppen in das FamiLinx-Sample wurden in der Literatur bereits erörtert. So dürfte die Überrepräsentation des „weißen Mannes“ weniger problematisch sein als in der globalen Betrachtung, da sich unsere Untersuchung auf das Deutsche Reich konzentriert. Die Übererfassung von Männern und Personen mit einem bestimmten Bekanntheitsgrad („notability bias“) verbleibt jedoch. Sind Letztere mit einem höheren sozialen Status verknüpft, ergibt sich eine erste Erklärung für die vermeintlichen Mortalitätsvorteile in FamiLinx. Stelter und Alburez-Gutierrez [8] dokumentieren dies anhand der klaren Überrepräsentation von Wissenschaftlern in FamiLinx. Allerdings beschränkt sich ihre Untersuchung auf die männliche Sterblichkeit. Ein Rückschluss auf die geschlechtsspezifische differenzielle Mortalität ist damit per Definition ausgeschlossen.

Eine Überrepräsentation von Eliten oder Individuen mit hohem Status sollte in der Familienrekonstitution keine Rolle spielen. Dafür erscheint die Dominanz von Orten in ländlichen Räumen – und das auch nur für einen kleinen Teil des Deutschen Reiches – ein möglicher Erklärungsansatz [3]. Allerdings würde der Fokus auf ländliche Räume tendenziell eine höhere (adulte) Lebenserwartung suggerieren, während die gefundenen Unterschiede relativ gering ausfallen.

Fanden die allgemeine Verzerrung der adulten Lebenserwartung und mögliche Ursachen in der Literatur bereits Aufmerksamkeit, rückte die geschlechtsspezifische Mortalität bisher kaum in den Fokus. Die vorliegende Untersuchung zeigt, dass Auswertungen von FamiLinx und der Familienrekonstitution kein verlässliches Abbild der in der amtlichen Statistik beobachteten differenziellen Sterblichkeit liefern. In FamiLinx und der Familienrekonstitution weisen Frauen im reproduktiven Alter höhere Sterberaten auf als Männer. Ein Phänomen, dass sich in der amtlichen Statistik nicht beobachten lässt. Angesichts der von den Verzerrungen insbesondere betroffenen Altersklassen erscheint ein Zusammenspiel von Müttersterblichkeit mit dem patriarchischen Ansatz bei der Erstellung von Familienstammbäumen ein naheliegender Erklärungsansatz. Die Dynastien werden häufig entlang der männlichen Nachkommen bzw. Vorfahren rekonstruiert. Das männliche Geschlecht ist, wie auch in unserem Teil des Samples von FamiLinx, überrepräsentiert. Hinzukommt die potenzielle Rolle der Müttersterblichkeit. Mit ca. 400 Sterbefällen auf 100.000 Lebendgeburten war die Geburt noch zum Ende des 19. Jahrhunderts, beispielsweise durch das Kindbettfieber, ein gesundheitlich riskantes Unterfangen für Frauen und die Müttersterblichkeit fernab von unbedeutend [24]. Kommt es nun zu einer Wiederverheiratung der Witwer, ergeben sich nicht wenige Lebensläufe eines Mannes mit mehreren früh verstorbenen Partnerinnen. Gleichzeitig werden früh verstorbene Männer oder Männer ohne Partnerinnen aufgrund fehlender Nachfahren weniger wahrscheinlich im Datensatz erfasst sein. Es kommt zur beobachteten Lücke in der Sterblichkeit in den mittleren Altersklassen.

Während die dominant patriarchische Erstellung von Familienstammbäumen und die Müttersterblichkeit plausible Erklärungsansätze für die Ergebnisse auf Grundlage von FamiLinx bieten, verbleibt die Frage nach den möglichen Ursachen für die Verzerrung in der Familienrekonstitution. Hier ist der Anteil der männlichen Bevölkerung deutlich geringer. Auch früh verstorbene Männer ohne Nachfahren sollten idealtypisch in einem Ortsfamilienbuch nicht untererfasst sein. Dafür tritt das Problem der räumlichen Beschränkung zutage. Auswandernde Individuen fehlen in der Betrachtung. Diese rechtszensierten Individuen fehlen bis zur Abwanderung in der Risikobevölkerung und die Sterberaten erscheinen höher, als sie tatsächlich waren. Wanderungen passierten häufig bis ins junge Erwachsenenalter, sodass insbesondere die Sterblichkeit bis zu diesen Altersklassen verzerrt wird. Die Korrektur durch Berücksichtigung der Bevölkerung bis zum letzten beobachteten demografischen Ereignis bietet eine Möglichkeit, die differenzielle Mortalität zu verbessern – ein Ansatz der beispielsweise in Imhof verfolgt wurde [18]. Eine Alternative bildet die bewusste Einbindung von Wanderungen in Ortsfamilienbücher mit der Ergänzung von Informationen für Ein- und Auswanderer. Diese könnte neben der Korrektur der Verzerrungen in den Daten mithilfe demografischer Methoden bereits im Ausgangsmaterial die Verzerrungen reduzieren.

Online-Genealogien bieten eine vielversprechende Alternative und Ergänzung zu den im Rahmen von Familienkonstitutionen gesammelten Daten. Eine Sterblichkeitsanalyse der Ahnentafeln in beiden Ansätzen als Approximation des Sterblichkeitsgeschehens der Allgemeinbevölkerung erfordert allerdings eine umfassende Validierung mithilfe von Referenzwerten, wie zum Beispiel der amtlichen Statistik. Während die generelle Untererfassung der Sterblichkeit in FamiLinx dominiert, konnte gezeigt werden, dass in beiden Datensätzen das wichtige Merkmal der geschlechtsspezifischen Sterblichkeitsunterschiede nicht korrekt abgebildet wird. Eine Interpretation der historischen Ergebnisse verlangt daher zwingend die gebührende Vorsicht.

## Footnotes

1 Die Verwendung von demografischen Ereignissen, wie z. B. Eheschließungen, um rechtszensierte Fälle in der Risikobevölkerung zu berücksichtigen, wird nicht vorgenommen. Damit ergeben sich nicht unerhebliche Abweichungen im Vergleich zur Arbeit von Imhof (siehe [18]).

2 Das Jahr 1871 würde demnach beispielsweise auf die Periode 1871–1880 verweisen.
